# Anatomical and structural brain changes throughout healthy aging in adults

**DOI:** 10.1002/alz70856_099690

**Published:** 2025-12-24

**Authors:** Maria Sevilla‐García, Federico Ramírez, Fernando Maestú

**Affiliations:** ^1^ Technical University of Madrid, Madrid, Madrid, Spain; ^2^ Center for Cognitive and Computational Neuroscience, Madrid, Madrid, Spain; ^3^ Complutense University of Madrid, Madrid, Madrid, Spain; ^4^ Department of Experimental Psychology, Cognitive Processes and Speech Therapy, Universidad Complutense de Madrid, Madrid, Madrid, Spain

## Abstract

**Background:**

Healthy aging is associated with anatomical and structural changes in the brain, which can impact cognitive function and neural plasticity. Understanding these changes is crucial to distinguishing normal aging from neurodegenerative processes and promoting healthy aging strategies.

**Method:**

We analyzed MRI data from 278 cognitive healthy adult participants ranging from 50 to 82 years old (mean age = 65.99 ± 8.13 years). All participants scored greater than 26 in MMSE and their cognitive health was assessed by a clinician. T1‐weighted images were processed with FreeSurfer to extract cortical and subcortical measures, namely hippocampal volume, cortical and subcortical gray matter volume and cortical mean thickness. We analyzed diffusion‐weighted data using MRtrix3 to assess structural connectivity across brain networks. Four DTI‐derived metrics were estimated: fractional anisotropy (FA), number of streamlines (NoS), axial diffusivity (AD), and radial diffusivity (RD). Finally, we examined the correlation between these metrics and age.

**Result:**

All anatomical measures showed a significant age‐related decline. Additionally, FA showed a significant decrease, while AD and RD exhibited significant increases with aging.

**Conclusion:**

Changes in anatomical and structural brain measures suggest that, even in the absence of pathological conditions, the brain undergoes significant changes with aging.

